# Comparison of Kato Katz, antibody-based ELISA and droplet digital PCR diagnosis of schistosomiasis japonica: Lessons learnt from a setting of low infection intensity

**DOI:** 10.1371/journal.pntd.0007228

**Published:** 2019-03-04

**Authors:** Pengfei Cai, Kosala G. Weerakoon, Yi Mu, Remigio M. Olveda, Allen G. Ross, David U. Olveda, Donald P. McManus

**Affiliations:** 1 Molecular Parasitology Laboratory, QIMR Berghofer Medical Research Institute, Brisbane, Australia; 2 Faculty of Medicine, The University of Queensland, Brisbane, Australia; 3 Department of Parasitology, Faculty of Medicine and Allied Sciences, Rajarata University of Sri Lanka, Saliyapura, Sri Lanka; 4 Research Institute for Tropical Medicine, Department of Health, Manila, Philippines; 5 Menzies Health Institute Queensland, Griffith University, Gold Coast, Australia; 6 icddr, b, Dhaka, Bangladesh; University of Florida, UNITED STATES

## Abstract

**Background:**

Zoonotic schistosomiasis in Asia, caused by *Schistosoma japonicum*, remains a major public health concern in China and the Philippines. The developing epidemiological and socio-economic picture of the disease in endemic areas necessitates the development of affordable and highly accurate field diagnostics as an important component in evaluating ongoing integrated control and elimination efforts.

**Methods:**

Three diagnostic methods, namely Kato-Katz (KK) stool microscopy, ELISA and droplet digital (dd) PCR assays, were compared by detecting infection in a total of 412 participants from an area moderately endemic for schistosomiasis in the Philippines.

**Results:**

This comprehensive comparison further defined the diagnostic performance and features for each assay. Compared with the ddPCR assay analysing DNA from faeces (F_ddPCR), which exhibited the highest sensitivity, the SjSAP4 + Sj23-LHD-ELISA had the best accuracy (67.2%) among all five ELISA assays assessed. Schistosomiasis prevalence determined by the SjSAP4 + Sj23-LHD-ELISA and ddPCRs was similar and was at least 2.5 times higher than obtained with the KK method. However, the agreement between these assays was low. In terms of cost and logistical convenience, the SjSAP4 + Sj23-LHD-ELISA represents a cost-effective assay with considerable diagnostic merits. In contrast, although the ddPCR assays exhibited a high level of diagnostic performance, the high cost and the need for specialized equipment presents a major obstacle in their application in screening campaigns.

**Conclusion:**

The SjSAP4 + Sj23-LHD-ELISA represents a cost-effective tool for the diagnosis of schistosomiasis that could prove an important component in the monitoring of integrated control measures as elimination draws closer, whereas the ddPCR assays, in addition to their high sensitivity and specificity, are capable of quantifying infection intensity. However, the high cost of ddPCR hinders its wider application in screening programs, although it could be a valuable reference in the development and improvement of other diagnostic assays.

## Author summary

Schistosomiasis japonica remains prevalent in China and in the Philippines. The current changes in the epidemiological and socio-economic picture in the endemic areas makes it imperative that affordable and more sensitive field diagnostics are developed as an important component to evaluate ongoing integrated control and elimination efforts. Three diagnostic approaches, namely Kato-Katz stool microscopy, ELISA and droplet digital PCR assays, were compared by detecting infection in a cohort from schistosome-endemic areas in the Philippines. This comprehensive comparison further defined the diagnostic performance and features for each assay. Prevalence of schistosomiasis determined by the SjSAP4 + Sj23-LHD-ELISA and ddPCRs was at least 2.5 times higher than that by the KK method. The prevalence determined by the SjSAP4 + Sj23-LHD-ELISA and ddPCRs was similar, but low agreements between these assays were evident. The ddPCR assays showed considerable diagnostic performance but the high associated costs and the need for specialized equipment present major obstacles in their application in screening campaigns although they can serve as reference standards for evaluating other diagnostic assays. The SjSAP4 + Sj23-LHD-ELISA represents a cost-effective tool for the diagnosis of schistosomiasis japonica and this assay could prove an important monitoring tool to evaluate the impact of integrated control measures over time.

## Introduction

Schistosomiasis japonica, a disease of poverty, remains a public health concern in China and the Philippines. A significant reduction in the prevalence of schistosomiasis has been achieved in China due to extensive integrated control efforts underpinned by mass drug administration (MDA) with the highly effective drug praziquantel [1, 2]. In China, the estimated number of human cases dropped from 840,000 in 2004 to 54,454 by the end of 2016 [3]. In the Philippines, an estimated 580,000 individuals were reported infected as of 2010 [4], and the prevalence in a number of endemic areas remains high although the intensity of infection has dropped in recent years [5, 6]. In this era of extensive MDA and as elimination is on the horizon, more sensitive diagnostic tools for diagnosis of schistosomiasis are required. For China, there is a need for improved diagnostic tools for effective surveillance and proof of elimination; for the Philippines, it is imperative to develop affordable and accurate field assays as an important component of integrated schistosomiasis control.

There are three major categories of methods available for schistosomiasis diagnosis: parasitological detection (e.g. the Kato-Katz (KK) method); serology, including antibody-detection (AbD) and antigen-detection (AgD); and molecular assays (e.g. circulating nucleic acids detection) [7, 8]. Stool examination (the KK method) has high specificity and remains the first-line diagnostic method for schistosomiasis; however, its sensitivity is insufficient in this post-MDA era due to the reduced prevalence and intensity of schistosome infections [8]. The enzyme-linked immunosorbent assay (ELISA) is a commonly used method for the screening of parasitic infections. Serological AbD methods based on crude extracted antigens (e.g. soluble egg antigen (SEA)) exhibit sufficient sensitivity but have a limited ability to discriminate past from active infections. Furthermore, these AbD methods exhibit high rates of antibody cross-reactivity with antigens from other helminths in infected individuals, a feature which is common in most schistosomiasis-endemic regions; recombinant antigen-based serological AbD assays improve on accuracy by reducing this cross-reactivity) [9]. Current AgD is based mainly on the detection of proteoglycan components known as circulating anodic antigens (CAAs) or circulating cathodic antigens (CCAs), which can be probed in serum and urine by ELISA or monoclonal-antibody-based lateral flow assays [10]. Yet, a recent study found that the rapid urine test (POC-CCA) produced a considerable false positive rate [11]. Molecular methods based on LAMP [12, 13] and PCR technology, including qPCR [14, 15], and droplet digital PCR (ddPCR) [16–18], provide alternatives with high accuracy and have been extensively developed for the in-house diagnosis of schistosomiasis, exhibiting considerable sensitivity and specificity [19]. However, they are rarely used in large-scale surveillance of schistosomiasis as a result of their higher cost compared with other methods. In the absence of a gold standard diagnostic test for elimination efforts, a comparison between available methods for the diagnosis of schistosomiasis is thus important, to further characterise the diagnostic features of each assay [9, 11, 20].

Previously, we developed in-house ELISA assays for diagnosis of *S*. *japonicum* infection based on the detection of IgG antibodies against the large hydrophilic domain of the 23 kDa Sj23 tegumental protein (Sj23-LHD), two saposin proteins (SjSAP4 and SjSAP5) and two combinations (SjSAP4 + Sj23-LHD and SjSAP5 + Sj23-LHD) [21] and established ddPCR assays to detect schistosome-derived DNA isolated from serum and fecal samples [18]. In the present study, we compared three diagnostic tools, the KK and our ELISA assays and ddPCRs, for detection of schistosomiasis in a human cohort from a moderate endemic area in the Philippines. Furthermore, other important aspects (i.e., equipment requirements, costs, and field application) for developing diagnostic tools against this neglected disease were also compared for these methods. The current study provides further diagnostic insights for antibody-based ELSA and ddPCR assays for diagnosis of schistosomiasis japonica.

## Materials and methods

### Ethical statement

Clinical samples (blood and feces) from the study participants in Palapag and Laoang, Northern Samar, the Philippines were collected, and the human research ethical approval for the study was granted by the Institutional Review Board of the Research Institute for Tropical Medicine (RITM), Department of Health, Manila, the Philippines (Number 2015–12) and the Human Research Ethics Committee, QIMR Berghofer Medical Research Institute (QIMRB), Brisbane, Australia (Ethics Approval: P524). All serum samples from healthy individuals were collected from Qiqihar, Heilongjiang Provence, China, and ethical approval was provided by the Ethics Committee of the Institute of Pathogen Biology, Chinese Academy of Medical Sciences, Beijing, China. Written informed consent was received from each study participant (for those aged less than 15 years, written informed consent was received from their legal guardians).

### Sample collection, processing, and storage

Clinical samples (feces and blood) were collected from 412 subjects from 18 barangays in Northern Samar, the Philippines, in 2015. All processed samples were stored at 4°C and transported on wet ice to RITM, where the samples were stored at -20°C. All samples were subsequently shipped to QIMRB, Australia on dry ice for further analysis. Individual stools (~10–15 g) were collected from each participant with ID-labeled fecal cups. Two fecal samples were sought from each individual on different days within a week for the KK test. The remainder of the first fecal sample (~10 g) was stored at 4°C, after fixing in 80% (v/v) ethanol, and used for DNA extraction. Blood samples (10 mL) were collected from each individual with id-labeled serum separation tubes (10-mL silica vacutainers). The blood samples were allowed to clot at ambient temperature for 30 min. After centrifuging at 1,500 *g* for 10 min, the serum samples were then collected. Serum samples of healthy individuals were obtained from Heilongjiang Province, a non-endemic area for schistosomiasis in China.

**Fig 1:**
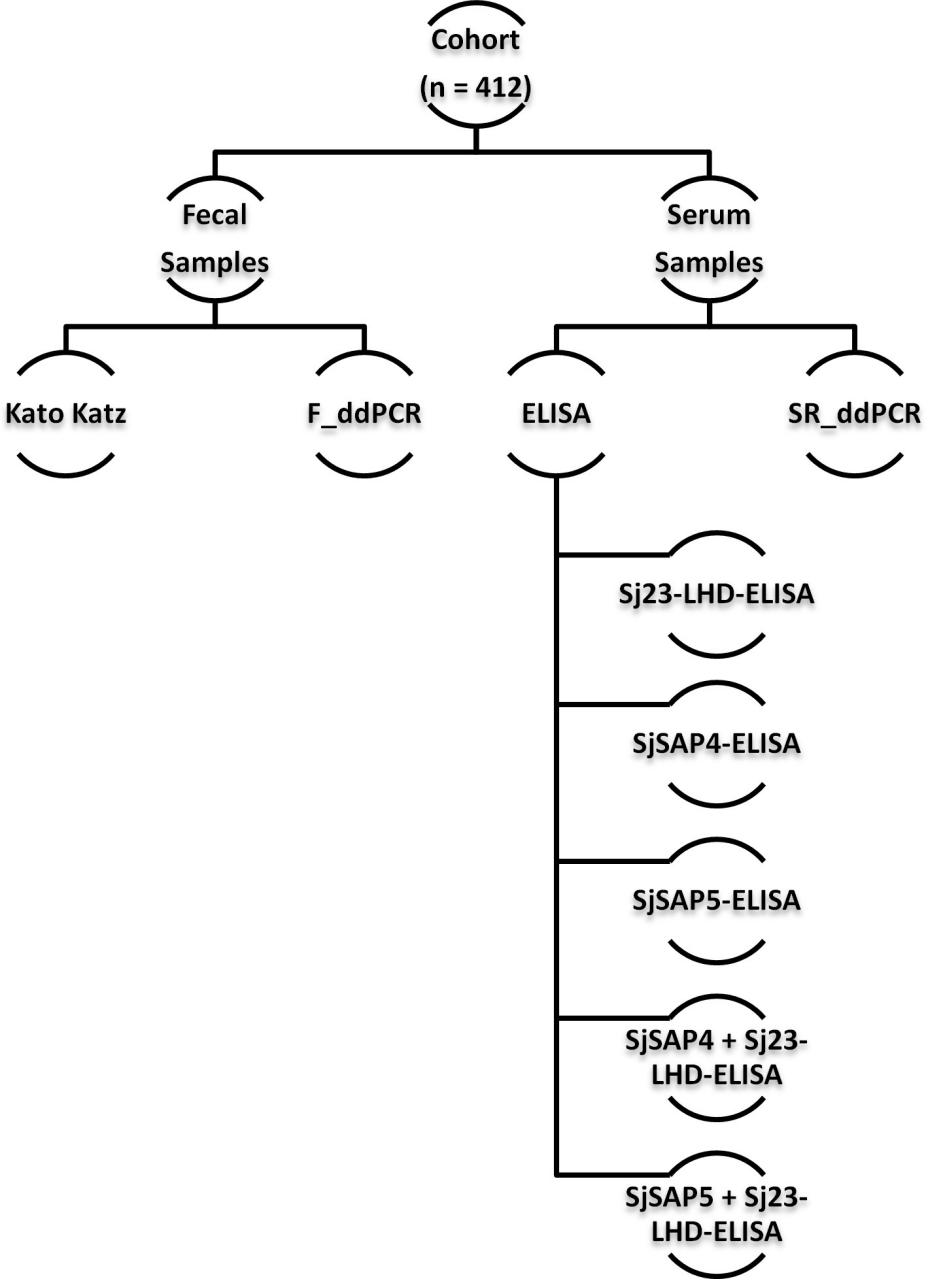
Flowchart showing the workflow for the detection of *S*. *japonicum* infection with different diagnostic methods in a cohort from an endemic area of Northern Samar, the Philippines. Fecal samples were examined with the KK technique, serum samples were tested by ELISA, while both samples were also analyzed by ddPCR.

Fig 1 shows the different diagnostic methods applied to stool and serum samples and the total number of samples analyzed by each parasitological test.

### Parasitological detection (Kato-Katz)

Individuals from the Philippine cohort were asked to provide two stool specimens from which 3 Kato-Katz thick smear slides were prepared for each sample. Slides were examined under a light microscope by experienced laboratory technicians. Infection intensity was presented as the number of eggs per gram of feces (EPG). For accuracy determination, 10% of slides were randomly selected and re-examined by an experienced microscopist.

### DNA extraction

Genomic DNA isolation of fecal samples was performed using the Maxwell 16 Instrument (Promega, Wisconsin, USA) incorporating the Maxwell 16 LEV Plant DNA kit. For each 200 mg of feces, 500 μL of ddH_2_O was added, mixed and the mixture centrifuged at 13,000 *g* for 5 min. After discarding the supernatant, 1 g of zirconia-silicate beads (0.5 mm) (BioSpec Products, Oklahoma, USA) and 500 μL of ROSE buffer were added. Samples were then homogenized (6,500 *g* × 40 seconds) using a Precellys tissue homogenizer (Bertin Technologies, Montigny-le-Bretonneux, France). The homogenate was incubated at 90°C for 10 min and centrifuged at 16,000 *g* for 5 min. The supernatant was transferred to a Maxwell 16 LEV Plant DNA kit cartridge, placed into the Maxwell 16 robot, and the plant DNA extraction protocol was then selected. Serum DNA extraction was performed using a ChemagicTM360 instrument (PerkinElmer Inc., Massachusetts, USA). 2 ml serum was used as an initial amount. DNA concentration for each sample was determined using a PowerWave HT microplate spectrophotometer (BioTek Instruments Inc., Vermont, USA). The fecal and serum DNA samples were diluted to 25 and 12.5 ng/μL, respectively, prior to ddPCR analysis.

### Droplet digital PCR analysis

The ddPCR was performed by amplifying an 82-bp fragment of the *nad1* gene [17, 18]. Briefly, the assay analyzed DNA extracted from feces (F_ddPCR) and serum (SR_ddPCR). Reaction mixture (20 μL) comprised 10 μL 1 × ddPCR EvaGreen master mix (Bio-Rad, Hercules), 1 μL primer pair (final concentration: 0.25 μM each), 2 μL template DNA, and 6 μL ddH_2_O. The reaction mixture was pipetted into a 96-well twin.tec plate (Eppendorf, Hamburg, Germany) and droplets were generated using an AutoDG instrument (Bio-Rad). The twin.tec plate was then sealed using a PX1 PCR Plate Sealer (Bio-Rad). PCR reaction was performed in a thermal cycler (C1000 Touch, Bio-Rad). The ddPCR cycling conditions were as follows: 95°C, 5 min, 40 cycles of 95°C for 30 sec, 60°C for 30 sec, and 72°C for 30 sec, and followed by a dye stabilization step at 4°C for 5 min and then 95°C for 5 min. Positive (using adult *S*. *japonicum* worm DNA as template) and negative (no DNA template) controls were used in all assays. Following PCR amplification, the plate was transferred to a QX200 Droplet Reader (Bio-Rad) for analysis. Fluorescence intensity of the negative control was used as the threshold for discrimination between droplets that contained target (positives) and those that did not (negatives), determined using QuantaSoft software version 1.3.2.0 (Bio-Rad) [16]. To calculate this threshold, every droplet was allocated to either a positive or negative cluster, and a proprietary method, based on Poisson-binomial statistical algorithms, was applied to the data to define the fluorescence threshold. This automatic threshold was manually adjusted for more stringent threshold limits within individual samples (higher than that of the negative controls).

### Evaluation of diagnostic candidates for schistosomiasis japonica by ELISA

The ELISA assay has been described previously [21]. Briefly, for the Sj23-LHD-, SjSAP4- and SjSAP5-ELISAs, all recombinant antigens were diluted to a final concentration of 1 μg/mL with coating buffer; for the SjSAP4 + Sj23-LHD- and SjSAP5 + Sj23-LHD-ELISAs, 50 ng of each antigen were mixed per well at 4°C overnight with 100 μL added per well. After blocking by blocking buffer (1% BAS in PBST) at 37°C for 1 h, Then serum samples diluted at 1:250 with blocking buffer were added (100 μL/well) and incubated for 1 h at 37°C. A mouse monoclonal anti-human IgG (Fc specific)-biotin antibody (Sigma-Aldrich Co, MO, USA) was used as secondary antibody (1:20,000, 100 μL/well), and samples were incubated for 1 h at 37°C. Streptavidin-HRP (BD Pharmingen, CA, USA) (1:10,000) was then applied to each well (100 μL/well). PBST washes were applied 5 times after each step, 2 min between each wash. Reactions were developed using TMB as substrate (100 μL/well) for 5 min and stopped using 2 M sodium hydroxide (50 μL/well). Optical density (OD) values were read at 450 nm using a microplate reader, and all tests were run in duplicate on each test plate.

### Statistical analysis

All results were input and stored in Microsoft Excel (2010) data base. The efficacy of specific anti-IgG antibodies for diagnosis was evaluated by the area under receiver operating characteristic (ROC) curve (AUC) (S1–S3 Figs). Cut-off values were set for ELISA assays with the maximization of Youden’s *J*-index (defined as *J* = Max_*c*_ {Se (*c*) + Sp (*c*) − 1}), using the KK, SR_ddPCR and F_ddPCR as references, respectively. Sensitivity, specificity, positive predictive value (PPV), negative predictive value (NPV) and accuracy were analyzed for each of other tests when compared to a reference test [22]. Sensitivity, specificity, PPV, NPV and accuracy were defined as follows: sensitivity = number of true positives / (number of true positives + number of false negatives); specificity = number of true negatives / (number of false positives + number of true negatives); PPV = number of true positives / (number of true positives + number of false positives); NPV = number of true negatives / (number of false negatives + number of true negatives); and accuracy = (number of true positives + number of true negatives) / (number of true positives + number of true negatives + number of false positives + number of false negatives). Statistical analyses were performed using GraphPad Prism version 7 (GraphPad Software, Inc., California). Agreement analysis between the KK, ELISA assays, and ddPCR tests were performed using the Kappa statistic with the online GraphPad software (https://www.graphpad.com/quickcalcs/kappa1/). The Altman’s benchmark scale was used to measure the strength of agreement according to the κ value, with the scores divided into: < 0.20 poor; 0.21–0.40 fair; 0.41–0.60 moderate; 0.61–0.80 good; 0.81–1.00 very good. Summary measures were expressed as means and 95% confidence intervals (CI).

## Results

### Characterization of the study cohorts

The parasitological study cohort comprised a total of 412 individuals, of which 218 were male (52.9%) and 194 were female (47.1%); the age range of participants was 5–69 years. The mean age of the cohort was 40.3 years (standard deviation = 15.7). Most individuals were in the 41–60 age range (n = 222, 53.88%), followed by those in the 21–40 age range (n = 102, 24.76%). For the healthy cohort, a total of 60 participants (29 (48.3%) males and 31 (51.7%) females, 26–60 years of age) were enrolled. The mean age (± SD) of the cohort was 42.5 ± 9.6 years.

### Egg burden determined by KK

**Fig 2:**
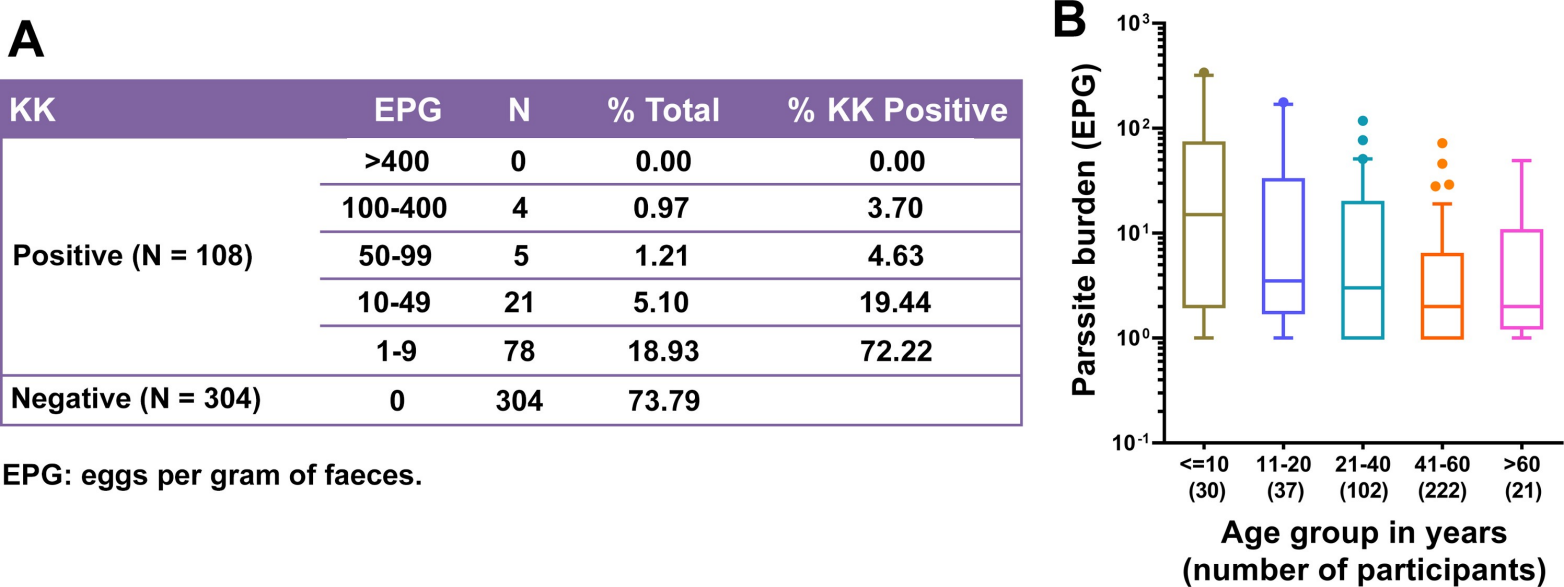
Classification of *S*. *japonicum* infection in the study cohort according to parasite load. A) Infection status and intensity determined by the KK method. B) Parasite load (EPG) in the different age groups. Boxes represent the interquartile range of the data with lines across the boxes indicating the median values. The hash marks above and below the boxes indicate the 90th and 10th percentiles, respectively. No statistically significant difference was observed between age groups (Non-parametric Kruskal-Wallis test, *p* > 0.05).

The fecal samples checked with the KK test (three slides) revealed that 108 individuals (26.2%) were positive for *S*. *japonicum* eggs (Fig 2A). The mean fecal *S*. *japonicum* egg burden in KK-positives was 17.6 ± 45.9 EPG. Among the KK-positives, the majority (n = 104, 96.3%) had a low parasite load of <100 EPG, and only 4 (3.7%) had a moderate infection, while none of them had a heavy infection (Fig 2A) according to the WHO categorization of schistosome infection severity. Infection prevalence and intensity were significantly higher in males than females based on the KK test [18]. Also, the intensity of *S*. *japonicum* infection was similar among individuals of different age groups (Fig 2B).

### Comparison of different methods for the diagnosis of schistosomiasis

Sensitivity, specificity, PPV, NPV, and accuracy were calculated for the KK, SR_ddPCR, F_ddPCR and SjSAP4 + Sj23-LHD-ELISA separately as the standard reference test. When using KK as the reference the SjSAP4-ELISA had the highest sensitivity (91.7%), but the lowest specificity (31.3%) and accuracy (47.1%) among the ELISA tests, while the F_ddPCR showed a higher sensitivity (98.1%) than the SR_ddPCR (94.4%), but had a lower specificity (33.9% vs 42.4%). The accuracy of the other tests ranged from 47.1% to 66.3%, while the agreement between the other tests and the KK reference standard showed a low concordance (κ < 0.3) (Table 1).

**Table 1:** Diagnostic performance of the different tests using the KK technique as reference.

Diagnostic test		KK		% Sensitivity (95% CI)	% Specificity (95% CI)	% PPV (95% CI)	% NPV (95% CI)	% Accuracy (95% CI)	Kappa Index (95% CI)
		+	-						
		N (%)	N (%)						
Sj23-LHD-ELISA	+	64 (59.3)	95 (31.3)	59.3 (49.4–68.6)	68.8 (63.2–73.9)	40.3 (32.6–48.3)	82.6 (77.4–87.1)	66.3 (61.5–70.8)	0.243 (0.149–0.337)
	-	44 (40.7)	209 (68.8)						
SjSAP4-ELISA	+	99 (91.7)	209 (68.8)	91.7 (84.8–96.1)	31.3 (26.1–36.8)	32.1 (27.0–37.7)	91.3 (84.2–96.0)	47.1 (42.2–52.0)	0.144 (0.092–0.195)
	-	9 (8.3)	95 (31.3)						
SjSAP5-ELISA	+	86 (79.6)	131 (43.1)	79.6 (70.8–86.8)	56.9 (51.1–62.6)	39.6 (33.1–46.5)	88.7 (83.4–92.8)	62.9 (58.0–67.5)	0.276 (0.197–0.354)
	-	22 (20.4)	173 (56.9)						
SjSAP4 + Sj23-LHD-ELISA	+	94 (87.0)	166 (54.6)	87.0 (79.2–92.7)	45.4 (39.7–51.2)	36.2 (30.3–42.3)	90.8 (85.0–94.9)	56.3 (51.4–61.2)	0.223 (0.157–0.290)
	-	14 (13.0)	138 (45.4)						
SjSAP5 + Sj23-LHD-ELISA	+	88 (81.5)	157 (51.6)	81.5 (72.9–88.3)	48.4 (42.6–54.1)	35.9 (29.9–42.3)	88.0 (82.1–92.5)	57.0 (52.1–61.9)	0.212 (0.140–0.283)
	-	20 (18.5)	147 (48.4)						
SR_ddPCR	+	102 (94.4)	175 (57.6)	94.4 (88.3–97.9)	42.4 (36.8–48.2)	36.8 (31.1–42.8)	95.6 (90.6–98.4)	56.1 (51.1–60.9)	0.245 (0.185–0.305)
	-	6 (5.6)	129 (42.4)						
F_ddPCR	+	106(98.1)	201 (66.1)	98.1 (93.5–99.8)	33.9 (28.6–39.5)	34.5 (29.2–40.1)	98.1 (93.3–99.8)	50.7 (45.8–55.7)	0.201 (0.152–0.250)
	-	2 (1.9)	103(33.9)						

Cut-off values for ELISA assays: Sj23-LHD-ELISA, 0.1537; SjSAP4-ELISA, 0.1168; SjSAP5-ELISA, 0.2921; SjSAP4 + Sj23-LHD-ELISA, 0.2394; SjSAP5 + Sj23-LHD-ELISA: 0.2046.

When using SR_ddPCR as the reference, the SjSAP5-ELISA exhibited the highest sensitivity (79.4%), but the lowest specificity (18.5%) within the ELISA assays, while the SjSAP4- and SjSAP4 + Sj23-LHD-ELISAs showed similar sensitivity as the SjSAP5-ELISA, but a relatively higher specificity (33.3% and 36.3%, respectively), thus showing higher accuracy (both 63.6%). The KK test had a low sensitivity of 36.8% but had the highest specificity (95.6%). The F_ddPCR showed the highest sensitivity (86.6%) and accuracy (74.8%) compared with any of the other tests with the SR_ddPCR as reference. The F_ddPCR and KK tests showed a fair (κ = 0.392 and 0.245, respectively) agreement with the SR_ddPCR, while all ELISA assays showed a poor (κ < 0.2) agreement with the SR_ddPCR as reference standard (Table 2).

**Table 2:** Diagnostic performance of different tests using the SR_ddPCR as reference.

Diagnostic test		SR_ddPCR		% Sensitivity (95% CI)	% Specificity (95% CI)	% PPV (95% CI)	% NPV (95% CI)	% Accuracy (95% CI)	Kappa Index (95% CI)
		+	-						
		N (%)	N (%)						
Sj23-LHD-ELISA	+	141 (50.9)	59 (43.7)	50.9 (44.8–56.9)	56.3 (47.5–64.8)	70.5 (63.7–76.7)	35.9 (29.4–42.7)	52.7 (47.7–57.6)	0.063 (-0.027–0.152)
	-	136 (49.1)	76 (56.3)						
SjSAP4-ELISA	+	217 (78.3)	90 (66.7)	78.3 (73.0–83.0)	33.3 (25.5–42.0)	70.7 (65.3–75.7)	42.9 (33.2–52.9)	63.6 (58.7–68.3)	0.124 (0.025–0.222)
	-	60 (21.7)	45 (33.3)						
SjSAP5-ELISA	+	220 (79.4)	110 (81.5)	79.4 (74.2–84.0)	18.5 (12.4–26.1)	66.7 (61.3–71.7)	30.5 (20.8–41.6)	59.5 (54.6–64.3)	-0.023 (-0.113–0.067)
	-	57 (20.6)	25 (18.5)						
SjSAP4 + Sj23-LHD-ELISA	+	213 (76.9)	86 (63.7)	76.9 (71.5–81.7)	36.3 (28.2–45.0)	71.2 (65.8–76.3)	43.4 (34.1–53.0)	63.6 (58.7–68.3)	0.138 (0.039–0.236)
	-	64 (23.1)	49 (36.3)						
SjSAP5 + Sj23-LHD-ELISA	+	182 (65.7)	84 (62.2)	65.7 (59.8–71.3)	37.8 (29.6–46.5)	68.4 (62.5–74.0)	34.9 (27.2–43.3)	56.6 (51.6–61.4)	0.034 (-0.063–0.131)
	-	95 (34.3)	51(37.8)						
KK	+	102 (36.8)	6 (4.4)	36.8 (31.1–42.8)	95.6 (90.6–98.4)	94.4 (88.3–97.9)	42.4 (36.8–48.2)	56.1 (51.1–60.9)	0.245 (0.185–0.305)
	-	175 (63.2)	129 (95.6)						
F_ddPCR	+	240 (86.6)	67 (49.6)	86.6 (82.1–90.4)	50.4 (41.6–59.1)	78.2 (73.1–82.7)	64.8 (54.8–73.8)	74.8 (70.3–78.9)	0.392 (0.297–0.488)
	-	37 (13.4)	68 (50.4)						

Cut-off values for ELISA assays: Sj23-LHD-ELISA, 0.1286; SjSAP4-ELISA, 0.1160; SjSAP5-ELISA, 0.1105; SjSAP4 + Sj23-LHD-ELISA, 0.1499; SjSAP5 + Sj23-LHD-ELISA: 0.1730.

When the F_ddPCR was employed as the reference, the SjSAP4-ELISA, the SjSAP4 + Sj23-LHD-ELISA and the SR_ddPCR showed a similar level of sensitivity (77.9, 76.5 and 78.2%, respectively); however, the SR_ddPCR had a higher specificity (64.8%) than all the ELISA assays, while the SjSAP4-ELISA and SjSAP4 + Sj23-LHD-ELISA showed a similar level of sensitivity as the SjSAP5-ELISA, but a relatively higher specificity (35.2% and 40.0%, respectively). The KK test exhibited 34.5% sensitivity and 98.1% specificity with F_ddPCR as reference, which was similar to that when using the SR_ddPCR as the reference. The SR_ddPCR exhibited the highest level of accuracy (74.8%) compared with any of the other tests. In regards to the ELISA assays, the SjSAP4 + Sj23-LHD-ELISA showed the highest accuracy (67.2%) and agreement (κ = 0.161), although had a poor concordance with the F_ddPCR reference (Table 3).

**Table 3:** Diagnostic performance of different tests using the F_ddPCR as reference.

Diagnostic test		F_ddPCR		% Sensitivity (95% CI)	% Specificity (95% CI)	% PPV (95% CI)	% NPV (95% CI)	% Accuracy (95% CI)	Kappa Index (95% CI)
		+	-						
		N (%)	N (%)						
Sj23-LHD-ELISA	+	157 (51.1)	43 (41.0)	51.1 (45.4–56.9)	59.0 (49.0–68.6)	78.5 (72.2–84.0)	29.2 (23.2–35.9)	53.2 (48.2–58.1)	0.076 (-0.006–0.159)
	-	150 (48.9)	62 (59.0)						
SjSAP4-ELISA	+	239 (77.9)	68 (64.8)	77.9 (72.8–82.4)	35.2 (26.2–45.2)	77.9 (72.8–82.4)	35.2 (26.2–45.2)	67.0 (62.2–71.5)	0.131 (0.029–0.233)
	-	68 (22.1)	37 (35.2)						
SjSAP5-ELISA	+	247 (80.5)	83 (79.0)	80.5 (75.6–84.7)	21.0 (13.6–30.0)	74.8 (69.8–79.4)	26.8 (17.6–37.8)	65.3 (60.5–69.9)	0.015 (-0.081–0.112)
	-	60 (19.5)	22 (21.0)						
SjSAP4 + Sj23-LHD-ELISA	+	235 (76.5)	63 (60.0)	76.5 (71.4–81.2)	40.0 (30.6–50.0)	78.9 (73.8–83.4)	36.8 (28.0–46.4)	67.2 (62.5–71.8)	0.161 (0.059–0.263)
	-	72 (23.5)	42 (40.0)						
SjSAP5 + Sj23-LHD-ELISA	+	207 (67.4)	59 (56.2)	67.4 (61.9–72.6)	43.8 (34.1–53.8)	77.8 (72.3–82.7)	31.5 (24.1–39.7)	61.4 (56.5–66.1)	0.100 (0.003–0.196)
	-	100 (32.6)	46 (43.8)						
KK	+	106 (34.5)	2 (1.9)	34.5 (29.2–40.1)	98.1 (93.3–99.8)	98.1 (93.5–99.8)	33.9 (28.6–39.5)	50.7 (45.8–55.7)	0.201 (0.152–0.250)
	-	201 (65.5)	103 (98.1)						
SR_ddPCR	+	240 (78.2)	37 (35.2)	78.2 (73.1–82.7)	64.8 (54.8–73.8)	86.6 (82.1–90.4)	50.4 (41.6–59.1)	74.8 (70.3–78.9)	0.392 (0.297–0.488)
	-	67 (21.8)	68 (64.8)						

Cut-off values for the ELISA assays: Sj23-LHD-ELISA, 0.1286; SjSAP4-ELISA, 0.1160; SjSAP5-ELISA, 0.1105; SjSAP4 + Sj23-LHD-ELISA, 0.1503; SjSAP5 + Sj23-LHD-ELISA: 0.1730.

When the most optimum ELISA assay, the SjSAP4 + Sj23-LHD-ELISA, was used as the reference, the SjSAP4-ELISA and SjSAP5 + Sj23-LHD-ELISA showed, respectively very good (κ = 0.832) and good (κ = 0.721) concordance with the reference. The F_ddPCR had a higher sensitivity (78.9%) than that of the SR_ddPCR (71.1%), but had a lower specificity (36.8% compared with 43.0%). Overall, the F_ddPCR showed a relatively higher accuracy (67.2% vs 63.3%) and concordance (0.161 vs 0.134) compared with the SR_ddPCR (Table 4).

**Table 4:** Diagnostic performance of different tests using the SjSAP4 + Sj23-LHD-ELISA as reference.

Diagnostic test		SjSAP4 + Sj23-LHD-ELISA		% Sensitivity (95% CI)	% Specificity (95% CI)	% PPV (95% CI)	% NPV (95% CI)	% Accuracy (95% CI)	Kappa Index (95% CI)
		+	-						
		N (%)	N (%)						
Sj23-LHD-ELISA	+	183 (61.4)	17 (14.9)	61.4 (55.6–67.0)	85.1 (77.2–91.1)	91.5 (86.7–95.0)	45.8 (38.9–52.7)	68.0 (63.2–72.4)	0.367 (0.289–0.446)
	-	115 (38.6)	97 (85.1)						
SjSAP4-ELISA	+	289 (97.0)	18 (15.8)	97.0 (94.3–98.6)	84.2 (76.2–90.4)	94.1 (90.9–96.5)	91.4 (84.4–96.0)	93.4 (90.6–95.6)	0.832 (0.771–0.893)
	-	9 (3.0)	96 (84.2)						
SjSAP5-ELISA	+	279 (93.6)	51 (44.7)	93.6 (90.2–96.1)	55.3 (45.7–64.6)	84.5 (80.2–88.3)	76.8 (66.2–85.4)	83.0 (79.0–86.5)	0.535 (0.441–0.629)
	-	19 (6.4)	63 (55.3)						
SjSAP5 + Sj23-LHD-ELISA	+	257 (86.2)	9 (7.9)	86.2 (81.8–89.9)	92.1 (85.5–96.3)	96.6 (93.7–98.4)	71.9 (63.9–79.0)	87.9 (84.3–90.9)	0.721 (0.650–0.792)
	-	41 (13.8)	105 (92.1)						
KK	+	99 (33.2)	9 (7.9)	33.2 (27.9–38.9)	92.1 (85.5–96.3)	91.7 (84.8–96.1)	34.5 (29.2–40.2)	49.5 (44.6–54.5)	0.167 (0.113–0.222)
	-	199 (66.8)	105 (92.1)						
SR_ddPCR	+	212 (71.1)	65 (57.0)	71.1 (65.6–76.2)	43.0 (33.8–52.6)	76.5 (71.1–81.4)	36.3 (28.2–45.0)	63.3 (58.5–68.0)	0.134 (0.035–0.232)
	-	86 (28.9)	49 (43.0)						
F_ddPCR	+	235 (78.9)	72 (63.2)	78.9 (73.8–83.4)	36.8 (28.0–46.4)	76.5 (71.4–81.2)	40.0 (30.6–50.0)	67.2 (62.5–71.8)	0.161 (0.059–0.263)
	-	63 (21.1)	42 (36.8)						

Cut-off values for ELISA assays: Sj23-LHD-ELISA, 0.1286; SjSAP4-ELISA, 0.1160; SjSAP5-ELISA, 0.1105; SjSAP4 + Sj23-LHD-ELISA, 0.1503; SjSAP5 + Sj23-LHD-ELISA: 0.1730.

### Prevalence analysis

**Fig 3:**
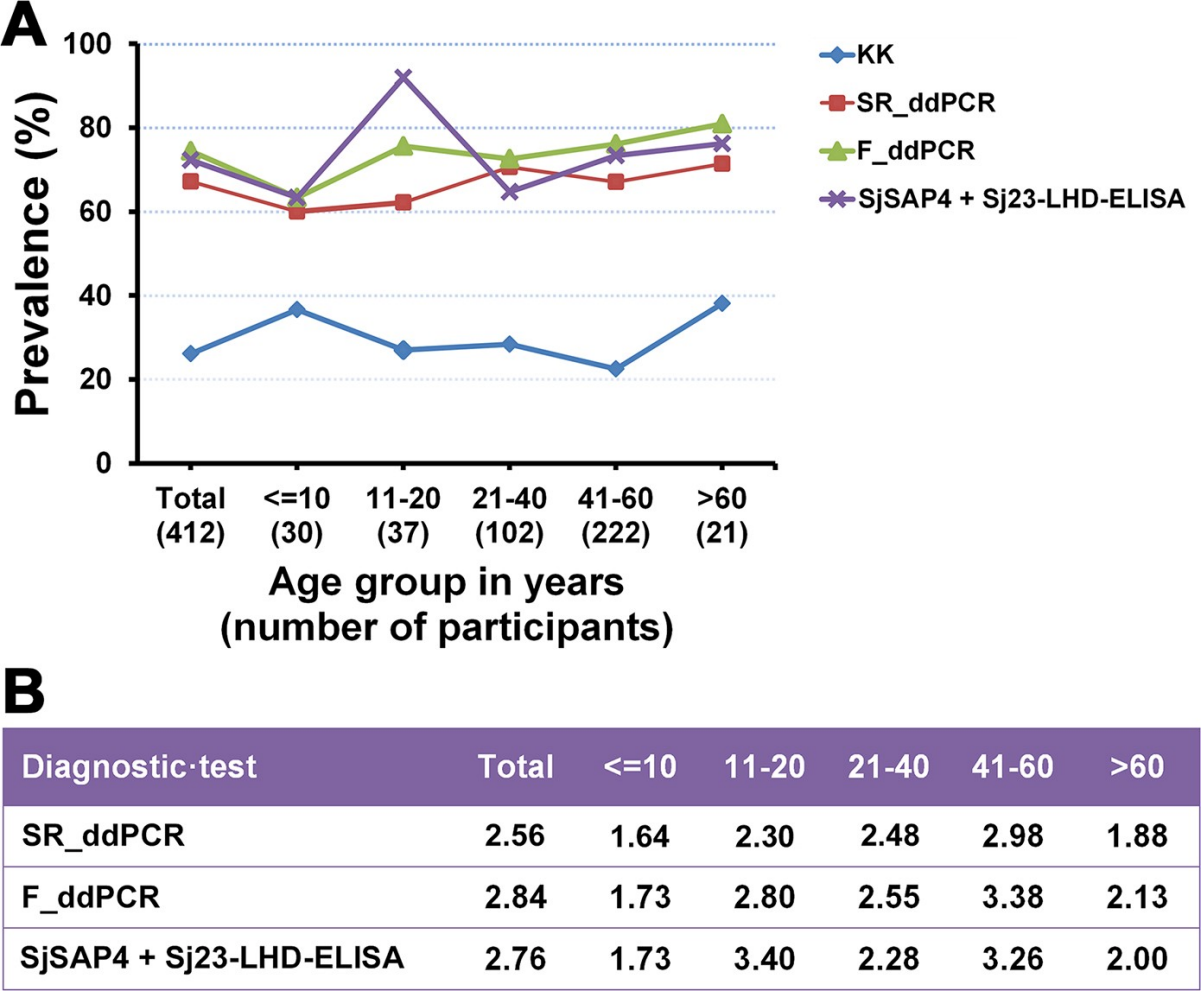
Comparison of prevalence of *S*. *japonicum* infection by different diagnostic tests. A) The prevalence of *S*. *japonicum* infection determined by the KK, SR_ddPCR, F_ddPCR and SjSAP4 + Sj23-LHD-ELISA for the total cohort by different age groups. B) Fold changes in the prevalence of *S*. *japonicum* infection determined by the SR_ddPCR, F_ddPCR and SjSAP4 + Sj23-LHD-ELISA vs the KK for the total cohort by each age group. Cut-off value for the SjSAP4 + Sj23-LHD-ELISA: 0.1503.

The prevalence of *S*. *japonicum* infection in the total cohort and different age groups determined by the KK method, SR_ddPCR, F_ddPCR and SjSAP4 + Sj23-LHD-ELISA is shown in Fig 3A. The lowest prevalence was observed in each age group by the KK, whereas with the other tests the infection prevalence increased with age except that the SjSAP4 + Sj23-LHD-ELISA showed the highest prevalence (91.9%) in teenage and young adults (11–20 years of age) (Fig 3A). The prevalence determined for each age group using the ELISA and the ddPCR tests was 1.64 to 3.40 times higher than the prevalence obtained with the KK method (three slides from one fecal sample) (Fig 3B).

### A comparison of other important features for the diagnostic methods tested

As shown in Table 5, we further compared other important aspects for developing the diagnostic tools in this study, including the amount of sample required, necessary equipment requirements, and the time required and the cost for each test.

**Table 5:** Equipment, time, cost and field accessible comparison of schistosomiasis diagnostic tests used in the study.

**Diagnostic test**	**Clinical sample**	**Amount**	**Equipment**	**Time** **(min/sample)**	**Cost**^#^ **(US$/sample)**	**Field application**
**KK**	stool	3 slides^1^	Light microscope	20^2^	0.05^2^	++
**ELISA**	serum	1 μL	Incubator Plate reader	2.5^3^	0.23	+
**SR_ddPCR**	serum	2 mL	Chemagic 360 DNA extraction system Thermal cycler QX200 Droplet Reader	6.0	11.57	-
**F_ddPCR**	stool	200 mg	Maxwell 16 Instrument Thermal cycler QX200 Droplet Reader	7.5	9.00	-

^#^Does not include equipment, infrastructure, and labor.

^1^For 3 slides per one stool sample.

^2^Estimated based on duplicate KK thick smear slides [23].

^3^Assuming five plates were used at a time.

## Discussion

China has attained substantial achievements in the control of schistosomiasis japonica, with elimination now on the horizon; however, the disease remains a major public health challenge in the Philippines with high prevalence reported in several endemic areas [4, 5, 24], although the intensity of infection has been reduced following extensive MDA implementation [6]. The continuation of this high prevalence is due, in part, to the pronounced rainfall levels in the schistosome-endemic areas, promoting propagation of the *Oncomelania hupensis* snail intermediate hosts, and also the large number of non-human mammalian reservoirs (particularly bovines; water buffaloes and cattle) which contribute significantly to disease transmission, which makes control more challenging and difficult [5]. Under this prevailing epidemiological setting, development of affordable and accurate diagnostic tools will be a lever to monitor disease control in the Philippines. In this study, we investigated the diagnostic performance of the KK and serological and molecular biological methods using samples collected from a parasitologically well-defined cohort from the Philippines.

By analysing the study cohort according to parasite load, and based on WHO criteria, the population was characterized as having moderate *S*. *japonicum* prevalence and low infection intensity. It is now recognised that egg detection in human stool does not reflect the true prevalence due to poor sensitivity, particularly in communities with low intensity infections [11, 20, 25]. This was again evident in the current study where the detection sensitivity of the KK was approximately 35% when the ddPCRs and SjSAP4 + Sj23-LHD-ELISA were used as references. In a previous study, when a reference integrating all positive results from any of 3 parasitological methods, including 18 KK slides, a saline gradient, and the Helmintex method (based on the use of magnetic beads to trap eggs in a magnetic field), was used, the estimated prevalence increased 2.3 times compared with the results obtained with two KK slides from one fecal sample [11]. Here, the estimated prevalence by SR_ddPCR and F_ddPCR was 2.56 and 2.84 times higher than that obtained with 3 KK slides in a cohort with much lower infection intensity, indicating a higher sensitivity was obtained with the ddPCR methods than that with the coproparasitological method. Also, when using KK as a standard, as well as analysing the sensitivity of different diagnostic tests for the detection of the infection stratified by the parasite load (S1 Table), we found that the recently developed SR_ddPCR and F_ddPCR methods showed a higher level of sensitivity than the ELISA assays. However, the most KK-positive but ELISA-negative individuals were in the group with very low egg burden (EPG 1–9) (S1 Table). Globally, the F_ddPCR exhibited the highest sensitivity among all the tests using the KK as reference, and it also showed appropriate ability in discriminating a past from an active infection since it primarily measures egg DNA, though it may also detect DNA from viable or dead eggs within a few weeks after a successful treatment; it can thus be expected to give a more accurate measure of prevalence when compared with the other tests. The estimated prevalence determined by the SjSAP4 + Sj23-LHD-ELISA (using F_ddPCR as a reference) was higher than that of the SR_ddPCR, but close to that of the F_ddPCR (67.2%, 74.5% and 72.3% for the SR_ddPCR, F_ddPCR and SjSAP4 + Sj23-LHD-ELISA, respectively), indicating some false positives with the ELISA, a recognized occurrence with serologically-based methods.

A high level of concordance was observed between the sensitive ELISAs, such as that between the SjSAP4-ELISA and the SjSAP4 + Sj23-LHD-ELISA (κ = 0.832) and between the SjSAP4 + Sj23-LHD-ELISA and the SjSAP5 + Sj23-LHD-ELISA (κ = 0.721). However, within the ddPCR assays, the concordance between the SR_ddPCR and the F_ddPCR was fair (κ = 0.392), which could likely be due to the different sample sources used for each assay, i.e., the SR_ddPCR detects cell-free DNA (cfDNA) released from different developmental schistosome stages (schistosomula, adults and eggs) in host serum, while the F_ddPCR primarily probes the DNA of schistosome ova in stool. It is noteworthy that although the prevalence of schistosomiasis determined by the SjSAP4 + Sj23-LHD-ELISA and ddPCRs were similar, the concordance between the ddPCR and the ELISA was poor (κ < 0.2), most probably due to the differences in biological targets detected by the two systems, i.e., the ELISA detects host IgG antibodies raised against target antigens, while the ddPCR detects schistosome-derived DNA in serum and/or stool samples. Furthermore, the low concordance between the ELISA and F_ddPCR might be explained by the fact that antibodies remain after the elimination of eggs in stool samples following chemotherapy, especially as a result of the following circumstances: 1) The endemic area has continuous all year round transmission of schistosomiasis but it is more pronounced during the rainy season, resulting in greater infection exposure; 2) The ongoing community-based chemotherapy (i.e., 40 mg/kg of praziquantel), initiated in the late 1980s, is only partially successful, due to low drug treatment coverage of those aged 5–65 years (<50%); 3) Reinfection is of high frequency, because most participants in the parasitological study cohort were engaged in rice farming, thereby having direct contact with contaminated water bodies [26]. In contrast, a reduced humoral immune response may occur in some individuals with an extremely low egg burden as has been observed in China [27]. On the other hand, since only a small amount of fecal sample was used for DNA extraction, as in the case with most fecal-based diagnostic methods, the F_ddPCR may not have detected infections due to the uneven distribution of eggs in the stool sample, the daily fluctuation of egg discharge, or by occult infections with just unisex [28] or aging worms [11]. In contrast to other age groups, the 11–20 years old group showed a relatively higher prevalence by the SjSAP4 + Sj23-LHD-ELISA compared with the SR_ddPCR and F_ddPCR, suggesting a relatively frequent exposure to infection, and/or a robust immune response elicited after infection in adolescents and a longer lasting level of specific antibody in this group.

Globally, although it still serves as a ‘gold standard’, the KK method, shown here also, is not sufficiently sensitive to identify the true infection status of individuals with low parasite burden [11, 29]. In contrast, the F_ddPCR represents a sensitive and specific diagnostic method. This tool is quantitative and showed a higher agreement with the KK based on intensity categories [18]. When using the F_ddPCR as a reference, the SjSAP4 + Sj23-LHD-ELISA and the SR_ddPCR exhibited a similar level of sensitivity (76.5% and 78.2%, respectively). For the ELISA, this may be explained by the fact that low antibody responders are present among schistosome egg-positive residents in low-transmission areas for *S*. *japonicum* as fore-mentioned [27]. For the SR_ddPCR, multiple developmental stages (schistosomula, adults and eggs) can be a source of the schistosome cfDNA in in serum or other body fluids [19]. Tegumental renewal [30], turnover [31], exosome secretion from live parasites and eggs, and decaying dead worms may liberate cfDNA into the blood. The relatively low sensitivity of the SR_ddPCR compared to the F_ddPCR probably results from the low concentration of schistosome cfDNA in serum when an individual harbors only a few worms; on the other hand, some serum samples may contain a high concentration of host-derived cfDNA, resulting in a relatively low percentage of parasite-derived cfDNA in the DNA samples tested in the SR_ddPCR. In terms of specificity, the SR_ddPCR showed a higher specificity (64.8%) than that obtained with the SjSAP4 + Sj23-LHD-ELISA (40.0%). As discussed previously, serological method for schistosomiasis diagnosis have limited ability to distinguish between ongoing and previous infections [9, 21]. By focussing on the subgroup of F_ddPCR-negative but SjSAP4 + Sj23-LHD-ELISA positive individuals (n = 63), analysis of the percentage of these individuals in each age group revealed that the highest was in the group of teenagers (11–20 years of age), which was not observed in the subgroup of F_ddPCR-negative but SR_ddPCR-positive individuals (S2 Table). This result echoed the suggestion in prevalence analysis that false positives are more readily found in adolescents by ELISA. Nevertheless, 24 individuals in the former subgroup were KK- or SR_ddPCR-positive, thus giving an estimated actual false positive (n = 39) in the ELISA using the F_ddPCR as the reference. To improve the ability to distinguish between live and past infections yet retain sensitivity, further optimization of the SjSAP4 + Sj23-LHD-ELISA is warranted. This may include adjusting the ratio of antigens in this combination ELISA, and detection of other antibody isotypes (such as IgM), in replacing some IgG levels as: 1) IgM detection will be beneficial for early diagnosis. 2) IgM usually has a short half-life; and 3) IgM will not be intensely elicited during reinfection [32]. However, the sensitivity of an IgM-ELISA using a combination of SjSAP4 and Sj23-LHD remains to be evaluated.

We further compared other important aspects for developing applicable diagnostics for detection of *S*. *japonicum* infection with particular focus on logistical convenience and the related operation costs. Of the serum-based assays, the SR_ddPCR was performed with an initial volume of 2 mL serum in reaching its optimum sensitivity, while 1 μL serum was sufficient for the ELISA. Hence, the ELISA would considerably encourage sampling compliance. With regards to the time involved, the KK takes about 20 minutes for the examination of three thick smear slides, while the ELISA, SR_ddPCR, and F_ddPCR take 2.5, 6 and 7.5 minutes, respectively, for testing one sample (Time consumption for serological and molecular assays are based on the total time required for a single high-throughput run). Thus, the KK is the most time-consuming method, followed by the F_ddPCR, SR_ddPCR and ELISA. Since schistosomiasis is endemic in developing countries, the costs involved are an important consideration when developing diagnostic tools for the disease. In terms of the materials, the KK, ELISA, SR_ddPCR and F_ddPCR cost 0.05, 0.23, 11.56 and 9.00 US$, respectively, for testing each sample. The high cost of the ddPCR assays poses a considerable challenge for their application in screening campaigns as is the case with most other molecular diagnostics [33]. Adding to this, the need of advanced and specialized equipment, coupled with the need for trained staff, makes the field application of the molecular methods even more challenging.

In conclusion, in areas of moderate endemicity (based on copro-parasitological testing) but low intensity infections, serological methods such as the SjSAP4 + Sj23-LHD-ELISA might prove sufficiently cost-effective to be included as additional complementary diagnostic procedures to the current KK method. Currently, the high cost of ddPCR presents a major obstacle against its application in widespread surveillance screening campaigns; nevertheless it could prove to be a valuable reference for serology-based and other diagnostic methods.

## Supporting information

S1 Fig Cut-off value determination for ELISA assays using the KK as a reference. (A) Scatter plots showing the IgG responses of healthy controls (n = 60) and KK-positives (n = 108) to Sj23-LHD, SjSAP4, SjSAP5, SjSAP4 + Sj23-LHD and SjSAP5 + Sj23-LHD, respectively, for the diagnosis of schistosomiasis japonica. KK-P: Kato Katz positive. (B) Receiver operating characteristic curve (ROC) analysis using the ELISA data of KK positives and healthy control.
(TIF)

S2 Fig Cut-off value determination for ELISA assays using the SR_ddPCR as a reference. (A) Scatter plots showing the IgG responses of healthy controls (n = 60) and SR_ddPCR positives (n = 277) to Sj23-LHD, SjSAP4, SjSAP5, SjSAP4 + Sj23-LHD and SjSAP5 + Sj23-LHD, respectively, for the diagnosis of schistosomiasis japonica. SR_ddPCR-P: SR_ddPCR positive. (B) ROC analysis using the ELISA data of SR_ddPCR positives and healthy control.
(TIF)

S3 Fig Cut-off value determination for ELISA assays using the F_ddPCR as a reference. (A) Scatter plots showing the IgG responses of healthy controls (n = 60) and F_ddPCR positives (n = 307) to Sj23-LHD, SjSAP4, SjSAP5, SjSAP4 + Sj23-LHD and SjSAP5 + Sj23-LHD, respectively, for the diagnosis of schistosomiasis japonica. F_ddPCR-P: F_ddPCR positive. (B) ROC analysis using the ELISA data of F_ddPCR positives and healthy control.
(TIF)

S1 Table Sensitivity of different diagnostic tests for the detection of schistosomiasis japonica considering the parasite load, as determined by egg counts of three Kato-Katz smear slides. (XLSX)

S2 Table Percentages of F_ddPCR negative but SjSAP4 + Sj23-LHD-ELISA positive and F_ddPCR negative but SR_ddPCR positive individuals in different age groups. (XLSX)
