# Supplementary figures and images for: Comparison of Kato Katz, antibody-based ELISA and droplet digital PCR diagnosis of schistosomiasis japonica: Lessons learnt from a setting of low infection intensity
Source: PLoS Negl Trop Dis. 2019 Mar 4;13(3):e0007228. doi: 10.1371/journal.pntd.0007228 (PMC6417743; doi:10.1371/journal.pntd.0007228)

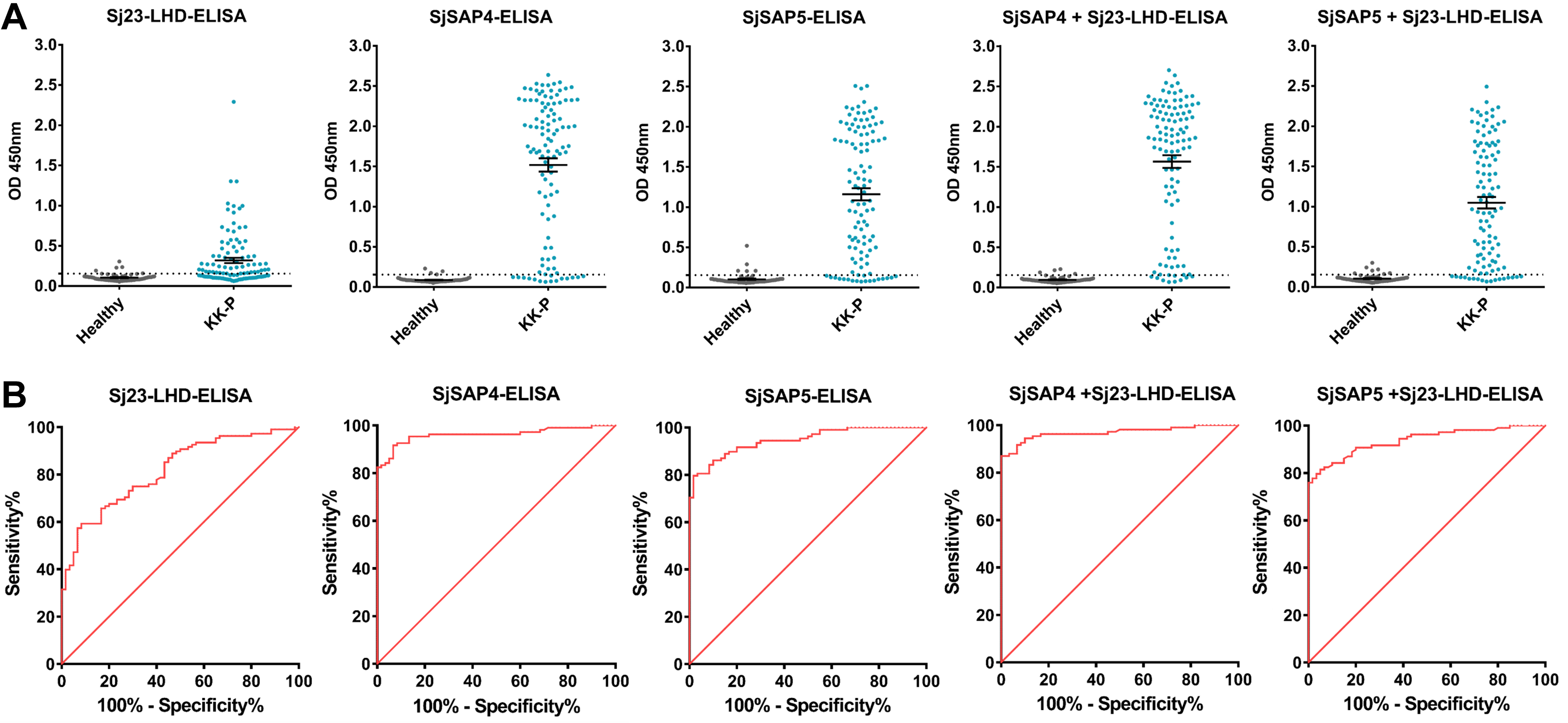

Supplement: S1 Fig — (A) Scatter plots showing the IgG responses of healthy controls (n = 60) and KK-positives (n = 108) to Sj23-LHD, SjSAP4, SjSAP5, SjSAP4 + Sj23-LHD and SjSAP5 + Sj23-LHD, respectively, for the diagnosis of schistosomiasis japonica. KK-P: Kato Katz positive. (B) Receiver operating characteristic curve (ROC) analysis using the ELISA data of KK positives and healthy control. (TIF) [file pntd.0007228.s001.tif]

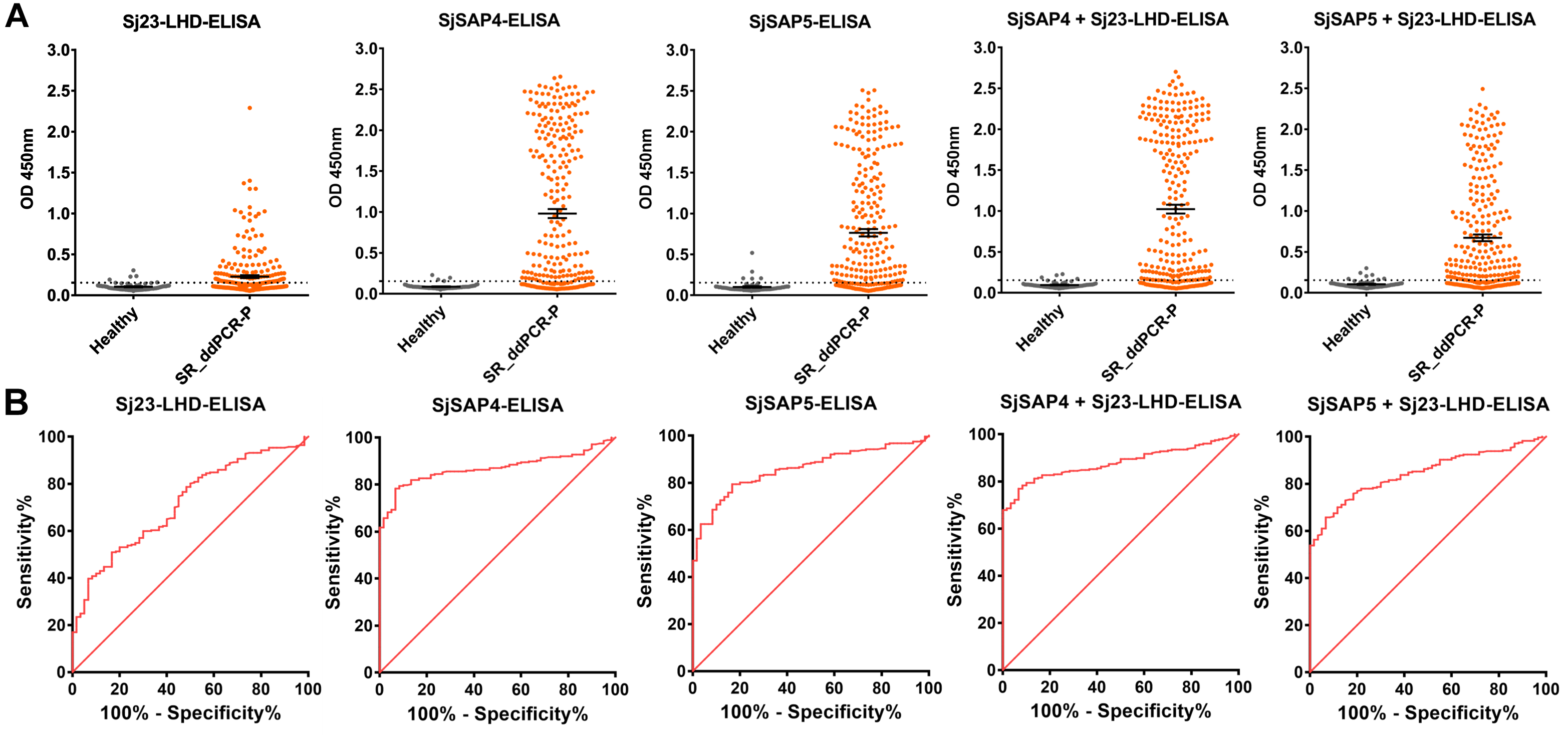

Supplement: S2 Fig — (A) Scatter plots showing the IgG responses of healthy controls (n = 60) and SR_ddPCR positives (n = 277) to Sj23-LHD, SjSAP4, SjSAP5, SjSAP4 + Sj23-LHD and SjSAP5 + Sj23-LHD, respectively, for the diagnosis of schistosomiasis japonica. SR_ddPCR-P: SR_ddPCR positive. (B) ROC analysis using the ELISA data of SR_ddPCR positives and healthy control. (TIF) [file pntd.0007228.s002.tif]

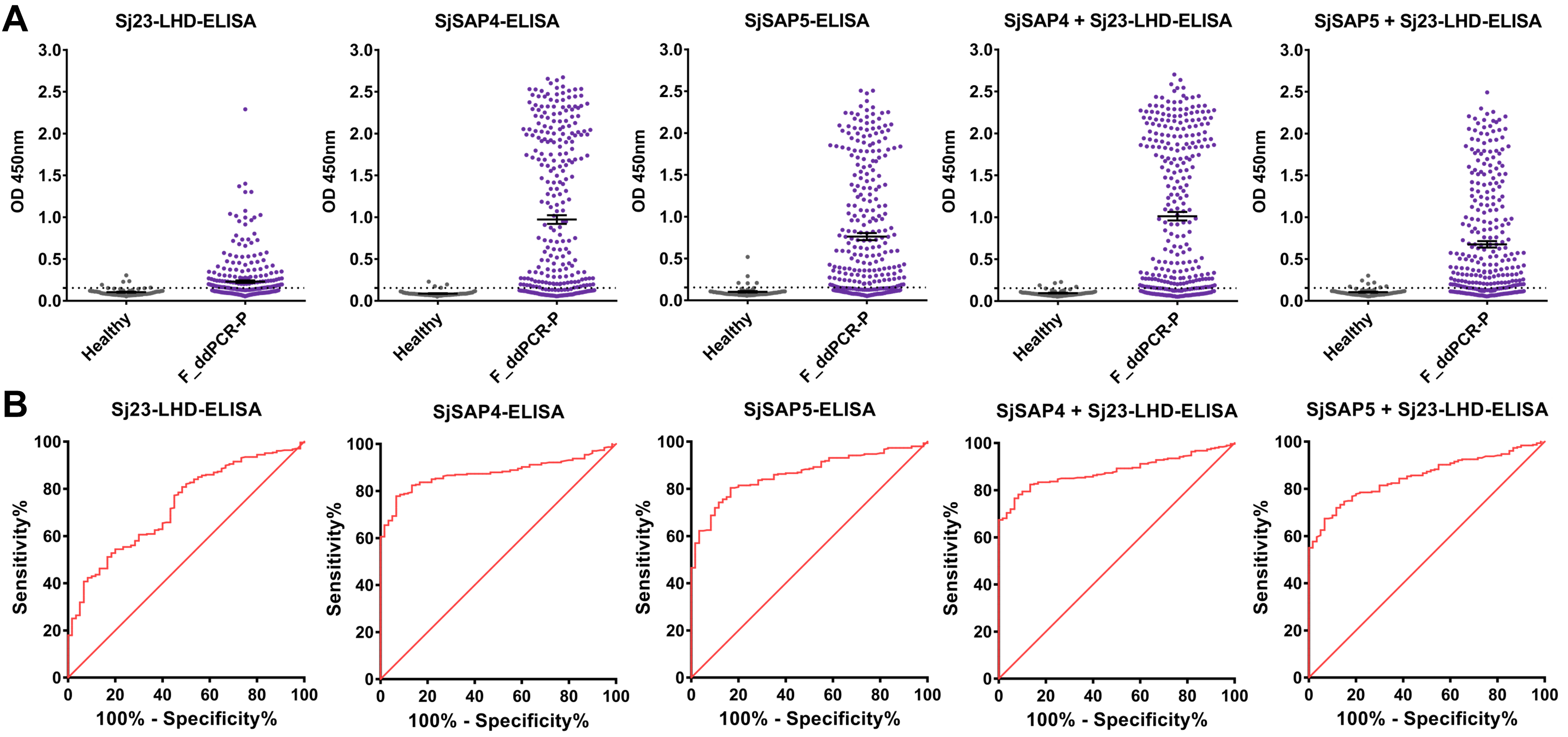

Supplement: S3 Fig — (A) Scatter plots showing the IgG responses of healthy controls (n = 60) and F_ddPCR positives (n = 307) to Sj23-LHD, SjSAP4, SjSAP5, SjSAP4 + Sj23-LHD and SjSAP5 + Sj23-LHD, respectively, for the diagnosis of schistosomiasis japonica. F_ddPCR-P: F_ddPCR positive. (B) ROC analysis using the ELISA data of F_ddPCR positives and healthy control. (TIF) [file pntd.0007228.s003.tif]
